# Service network analysis for agricultural mental health

**DOI:** 10.1186/1472-6963-9-87

**Published:** 2009-05-29

**Authors:** Jeffrey D Fuller, Brian Kelly, Susan Law, Georgia Pollard, Lyn Fragar

**Affiliations:** 1 Department of Rural Health, University of Sydney and Southern Cross University, Lismore, NSW, Australia; 2 Centre for Rural and Remote Health, University of Newcastle, Orange, NSW, Australia; 3 Australian Centre for Agricultural Health and Safety, University of Sydney, Moree, NSW, Australia; 4 North Coast Area Health Service, NSW, Australia

## Abstract

**Background:**

Farmers represent a subgroup of rural and remote communities at higher risk of suicide attributed to insecure economic futures, self-reliant cultures and poor access to health services. Early intervention models are required that tap into existing farming networks. This study describes service networks in rural shires that relate to the mental health needs of farming families. This serves as a baseline to inform service network improvements.

**Methods:**

A network survey of mental health related links between agricultural support, health and other human services in four drought declared shires in comparable districts in rural New South Wales, Australia. Mental health links covered information exchange, referral recommendations and program development.

**Results:**

87 agencies from 111 (78%) completed a survey. 79% indicated that two thirds of their clients needed assistance for mental health related problems. The highest mean number of interagency links concerned information exchange and the frequency of these links between sectors was monthly to three monthly. The effectiveness of agricultural support and health sector links were rated as less effective by the agricultural support sector than by the health sector (p < .05). The most highly linked across all areas of activity were Rural Financial Counsellors, the Department of Primary Industry Drought Support Workers and Community Health Centres. Hence for a mental health service network targeting farming families these are three key agencies across the spectrum of case work to program development. The study limitations in describing service networks relate to the accuracy of network bounding, self report bias and missing data from non participants.

**Conclusion:**

Aligning with agricultural agencies is important to build effective mental health service pathways to address the needs of farming populations. Work is required to ensure that these agricultural support agencies have operational and effective links to primary mental health care services. Network analysis provides a baseline to inform this work. With interventions such as local mental health training and joint service planning to promote network development we would expect to see over time an increase in the mean number of links, the frequency in which these links are used and the rated effectiveness of these links.

## Background

Farmers represent a subgroup of rural and remote communities at higher risk of various health problems [1,2]. Suicide, in particular, continues to be a major cause of death by injury among farmers in Australia [3]. The health risk of farmers is influenced by their occupational, environmental and social conditions, which includes poor access to health care services in rural and remote areas in general [4], and by their own specific cultural barriers to health care [5,6]. Financial and business pressures in agriculture in recent years have compounded isolation and have added further economic disincentives to these barriers in access to health services [3,5].

The current very prolonged drought affecting large areas of south-eastern Australian has contributed accumulated adversity to rural and remote communities, especially those most dependent on agriculture. The attendant socio-economic strain and social impact of drought for people in farming [7] highlights the need to consider ways to effectively meet their mental health needs [8]. An important improvement in access to mental health care is suicide prevention and the most effective strategies for suicide prevention include those that improve access to appropriate services (such as health services) and improve the quality of response received from those services [9].

A focus on potentially unmet mental health needs requires close consideration of the existing patterns of help-seeking. Caldwell et al [10] report that there are fewer presentations to primary care clinicians for mental health problems in rural areas than in urban areas. Self-reliant characteristics, attitudes towards mental health problems and perceived stigma are claimed to inhibit help seeking from professional mental health services. Australian data suggest that many common forms of distress experienced by farmers can be attributed chiefly to financial pressures and trusted support workers for whom no stigma-related barriers exist, such as rural financial counsellors, have been identified as important initial contact points for help [11-13]. While these workers have the specific role of assisting farmers in managing financial issues (such as seeking drought-related assistance & negotiating benefits), they can play a key role in improving their clients' access to health services. Rural financial counsellors have identified the emotional distress of their clients as a major concern. They have also identified their own need for improved skills in recognising and responding to this distress, and for improved local referral pathways [12].

The inclusion of "front-line" agricultural workers in the broad health care network of a rural community is warranted in view of their role as an important and trusted source of advice to farmers. A strategy should identify the range of services available in a community and develop clear local pathways to care. Such an approach supports health services personnel to recognise the role that other "front-line" agencies can play in the broader tasks of gaining care and completing recovery. This recognition includes identifying the benefits of on-farm contact and the support that can be provided from community organisations, such as the rural financial counsellors, in assisting with financial stressors that contribute to mental distress [14].

Based on earlier work that established the "front line" human service contact of agricultural support agencies, the aim of the study was to describe the mental health service links between these agencies and local health and human services [12]. This description would establish baseline measures of strength, weakness and opportunity in the local service network that could then inform a subsequent network improvement strategy.

## Methods

Four shires in rural New South Wales were purposively selected that had not recently received any mental health service development strategy. Shires were chosen between a population size of 3000 and 20,000 so they would be large enough to have a service network but not so large a network to make the analysis unmanageable. Our experience in a pilot study was that a network size of up to 30 agents was satisfactory for descriptive network analysis, whereas a larger network would make data collection quite resource intensive when this was being done by interview [15].

We adapted a bounded network analysis technique from Provan et al who used this method to both describe and then inform the improvement of local community service partnerships [16]. This technique surveys each member of a defined network about their links with all other members in that network. Two sets of measures are generated, measures which each member reports about their links with others (that member's OutDegree), and those which all others report about their links with that member (that member's InDegree). For more information about network analysis the article by Hawe at al provides an easy to read introduction [17].

The technique first required the construction of a relevant agency list for each shire (the bounded network) as these were the agencies that each were surveyed about. Constructing a bounded network list is relatively easy for defined groups, such as a classroom or partnerships where formal agreements exists, where people or agency names are recorded and where membership is clearly defined. In a local community health network, where some relationships may be informal, that may occur opportunistically and where members may change, constructing such a list is not so clear cut. In order to maximise capture of the relevant agencies in each shire on a list, we identified three knowledgeable key informants per shire from different service sectors. We asked them which other agencies they considered had some involvement in the mental health related needs of farming families, either through individual case work, group work or community and agricultural development. We prompted these key informants to consider agencies from the three sectors of agricultural support, health (mental health & other health) and other human services. Using three key informants from different service sectors maximised the spread of other agencies listed and minimised a biased focus on any one sector.

Agencies were delimited to those with a physical presence in the shire, meaning that services such as statewide telephone based support agencies were not included. A final agency list per shire was created as an amalgam of what the three informants described plus a check against what we researchers theoretically considered should be listed (ie the local GP, the nearest mental health team etc). While this did not eliminate the possibility that an agency would have been left off the list, the process did minimise this possibility. Each of the listed agencies was approached and interviewed through the most senior relevant service worker. As the agencies differed in size and function (some were one person operations and others much larger), then the actual agency informants varied from the sole service provider in some cases to the local service manager in others.

The survey involved standardised administration by interview of an instrument that covered interagency links to do with the following activities: (1) information exchange about providing mental health related help to clients, (2) recommending clients to other agencies for further help, and (3) working together in other ways (than casework) such as on advocacy or program development. Survey questions asked informants to recall the presence of a link, the frequency in which a link was used and the effectiveness of that link. Rather than specify a recall timeframe (say the last 3 months) we simply asked informants to recall whether they linked with a listed agency, then "in general" how often they linked and how effective they rated that link. This approach is consistent with the methodological literature that suggests informant recall is more reliable with typical events over general timeframes than specific events over defined timeframes [18]. The survey instrument was adapted from that used by Provan at al, with changes to reflect our context which we had tested for face validity in an earlier pilot study in two other shires [15]. The pilot study helped us to refine the wording of our questions and the associated definitions that we included in order to minimise uncertainty and ambiguity about what was being asked. Reliability was also increased by the use of only two interviewers who were trained together in the survey administration. The survey instrument is included as Additional file 1.

Data were entered into the UCINET v6 program for descriptive analysis and then exported to STATA v8 for further analysis [19,20]. The mean number of agencies that were linked was calculated per activity per shire, with the UCINET program applying a normalisation algorithm. This normalisation enables networks of different size to be compared, otherwise difference in means could simply be an effect of the different network size. Across the four shires overall we tested the difference in the mean links per activity (using one way ANOVA). Then for the scaled data (frequency and effectiveness scores) we extracted the scores from all of the four shires that were either agriculture agencies reporting links to health agencies or vice versa. Where the scale could be considered interval (effectiveness) then we calculated an overall mean score per activity, and where only ordinal (frequency) then we calculated medians. To test for significant difference between these scaled data we used the non-parametric Mann Whitney test because the data were either skewed or ordinal. We then analysed the network position of specific agencies per shire by allocating a rank score from the most highly linked (score 1) to the least linked and then to get an overall score we calculated the median of each agency's rank scores across the four shires. This enabled us to identify which agencies were the highly and least connected in the local rural mental health service network for farmers.

The University of Newcastle Human Research Ethics Committee approved the study. The surveys were conducted between May to October 2007.

## Results

Four shires were identified across comparable agricultural regions of New South Wales and all were drought declared at the time of the interview. Table 1 outlines the characteristics of these communities (eg population). Because we wanted to establish a baseline prior to the testing of a network improvement strategy (to be reported in later papers), we selected those shires that had not yet received any of the Mental Health First Aid training that was progressively being provided through various national and state government mental health responses to the drought [21].

**Table 1:** Shire details

	**D**	**E**	**F**	**G**
population	6400	13100	6500	10138

drought declared	Yes	Yes	Yes	Yes

location (relative to Sydney)	400 kms SW	300 kms W	600 kms SW	600 kms W

The shire population size ranged from 6000 to 13,000 and they were located between 300 to 600 kilometres from Sydney (the state capital).

Between 24 to 32 agencies were identified per shire with 21 to 22 per shire agreeing to participate. Of the 111 agencies identified, 87 completed a survey (78%). While there was variability within each shire, the distribution of interviewed agencies overall across the three agency types was fairly even, with 27 agricultural support agencies, 28 health and 32 other human service agencies (table 2). Hence, potential bias of a particular service sector is lessened when we aggregated data from across the four shires.

**Table 2:** Agency category details

**Agency type**	**D**	**E**	**F**	**G**	**total**
agricultural support	5	7	7	8	27

health	9	6	8	5	28

other human services	8	9	6	9	32

total interviewed	22	22	21	22	87

bounded list size	32	24	31	24	111

### Need

Seventy nine percent (79%) of the interviewed agencies indicated that at least two thirds of their clients were in need of assistance for mental health related problems and most agencies (77%) indicated that they do recommend that farming clients see other workers for help in these situations. Well under a half (38%) indicated that they meet regularly as a group or network with other agencies in their shire and this varied per shire from between 33–45%.

### Agency links per activity

There was no consistency between the shires on which activity had the highest number of linked agents and there was no statistically significant difference, the overall ordering was from information exchange, then making recommendations to working together in other ways (table 3).

**Table 3:** Activities: normalised mean link scores

	**D**	**E**	**F**	**G**	**overall mean**
info give	17.24	18.11	16.98	19.74	18.02

info receive	12.6	17.75	13.54	20.1	15.99

recommend	10.35	19.2	14.51	20.29	16.09

work together	14.92	17.93	8.38	16.66	14.46

### Frequency & effectiveness of links

We were particularly interested in the links between the agricultural support sector and the health sector and so we examined the frequency and effectiveness that each sector rated their links with each other (tables 4 and 5).

**Table 4:** Overall median frequency scores

	**ag to hlth**	**hlth to ag**	**ag to OHS**	**OHS to ag**
give info	3	4	3	4

rec info	3	4	3	3.5

recommend	3	4	3	4

work together	3	4	4	3

Data summarised using OutDegree rather than in other sections where InDegree used.

Scale 5 = weekly; 4 = monthly; 3 = three monthly; 2 = half yearly; 1 = less often that half yearly

Significant difference calculated using Mann-Whitney Test

**Table 5:** Overall mean effectiveness scores (agricultural support & health)

	**ag to hlth – mean (n)**	**hlth to ag – mean (n)**	**p**
give info	3.04 (10)	4.2 (15)	0.007

rec info	3.5 (12)	4.31 (13)	0.057

work together	3.65 (12)	4.10 (14)	0.32

Data summarised using OutDegree rather than in other sections where InDegree used.

Scale 5 = very good; 4 = good; 3 = neither; 2 = poor; 1 = very poor

Significant difference calculated using Mann-Whitney Test

Overall the median frequency in which the reported links were used between the agricultural support and health and other human services was between monthly to three monthly and there was no significant difference found between sectors across all of the activities.

In terms of effectiveness, the agricultural support sector rated giving information as significantly less effective than did the health sector. This difference was similar for receiving information although the significance was just outside the conventional level of probability. Effectiveness data on making recommendations was not collected because informants may not have known if a client acted on a recommendation and so could not rate effectiveness of this. There was no statistically significant difference between the agricultural support and health sector in the effectiveness rating of working together, although the trend here was the same, with the agricultural sector giving a less effective rating than did the health sector.

### Main agencies

Across all activities the highest linked agency came from agricultural support (the rural financial counsellors) with the Department of Primary Industry drought support workers and the community health centres the second highest linked. The community mental health teams, the GPs and the Centrelink rural support officers were relatively well linked at fourth and fifth highest (table 6).

**Table 6:** Rank link scores across all activities (all shire aggregated)

	**g_info**	**r_info**	**rec**	**w_tog**	**median rank**	**multiplexity**
Catchment Management Authority	8	7	8	7	7.5	0.40

Centacare	6	6	5	6	6	0.80

Centrelink (local office)	5	4.5	3.5	5.5	4.75	0.85

Centrelink Rural Support Officer	4	4	5	4	4	0.78

Community Health Centre	3	3	3	4	3	1.03

Dept Community Services	7	6	6	9	6.5	0.50

DPI Drought Support Worker	3	3	4	2	3	1.05

Drought MH Assist Prog/Liaison	7	5		4.5	5	0.49

Farmers Assoc	7	6	8	4	6.5	0.73

GP	3	5	3	8	4	0.77

Hospital	7	5	8	7.5	7.25	0.44

Community Mental Health Team	3	4	3	5	3.5	0.93

Psychologist/Counsellor	6	6	6	6	6	0.49

Rural Assistance Authority	7	7	7	6	7	0.59

Rural Financial Counsellor	1	1	1	1	1	1.58

Rural Lands Protection Board	6.5	5.5	6.5	4.5	6	0.61

Salvation Army	6	5	6	8	6	0.70

St Vincent de Paul	4	6	5	5	5	0.64

Agencies only included if ranked in at least 3 shires – hence some rank scores missing. Rank scores: 1 = highest linked

As well as the number of links, the strength of an agency in the network can also be measured by the extent in which their overall relationship with others is embedded. For instance an agency that is linked to another on all activities will have their overall relationship maintained even if some of those activities were to cease. This embeddedness is measured by the multiplexity score, and in this network the highest possible score is 4 (number of activities). A score of 4 would indicate that an agency was linked to all other agencies on all activities. The agencies with the highest scores were rural financial counsellors (1.58), the Department of Primary Industry drought support workers (1.05) and the community health centres (1.03).

## Discussion

The extent of need evident in agency responses does indicate that mental health issues were prominent in these four shires, which includes responses from some agencies for whom we would not have considered mental health as core business. This does support our rationale that developing a local service network that includes this range of agencies could improve both access to and the quality of mental health care in rural locations.

The most highly linked and multiplex status of the rural financial counsellors indicates that they were the most prominent and embedded (in network terms) as service providers and as intermediaries in mental health service networks for farming families. This prominence and embedded status is consistent with their reported trusted helper status and also signifies a probable financial and a counselling component of farmers mental health need. This contact with rural financial counsellors offers a window of opportunity for early intervention links that could form an important part of suicide prevention for this group. The other prominent and embedded agencies were the DPI drought support workers and community health centers. Our earlier work identified that rural financial counsellors do want better links with mental health and other social counsellors, training in mental health issues and also the development of appropriate and effective referral processes to health professionals [12].

As agencies to whom information was given and agencies to whom clients were recommended, general practitioners were the most prominent after the rural financial counselors, and equal with community centres, the mental health teams and drought support workers. However, general practitioners were not as highly linked on working together in other ways as would be expected given their clinical focus. Of interest is the relative high link status of the Farmers Association in working together in other ways and it is in this activity and through such agencies where community mental health promotion might occur. The relatively low status of the Drought Mental Health Assistance Program might be explained by the recency of this program and also by the non-clinical role of program workers. The status of this program might be expected to increase over time, particularly on working together in other ways.

The lower rating of linkage effectiveness by the agricultural support sector does make sense given that agricultural support workers are not trained in mental health and so would be expected to rate their communication about mental health issues as less effective. However, this finding, which is about effectiveness of linking with the health sector, could also reflect a dissatisfaction of rural communities with their access to mental health services in general [22,23].

Given the potential to include agricultural support agencies in a local mental health service network and also the indicators of room for improvement, we suggest that strategies to increase local capacity are warranted. This capacity could include early recognition of mental health distress, first level responding skills and knowledge about and confidence in referral options to professional mental health care. Mental Health First Aid training is a proven program to increase recognition skills and through this current research we are studying whether networking strategies, that include a community mental health development worker along with service network meetings increases referral capacity [24]. Such developmental work around early recognition rather than crisis responding would require local service planning to establish the different capacities and potential functions of local agricultural support and human service agencies and this work could come under the auspice of community mental health services. Given that financial issues faced by agricultural communities may also lead to family tension and relationship problems, then a network that provides family support and social counselling would be ideal, as well as those services for people who have developed a mental illness. With a range of agencies in a network, then a range of services can be provided, and in a more coherent and coordinated way. Agencies such as the church based welfare organisations (Centrecare), community health centres and psychologists do currently provide counselling services and they do join with agricultural agencies to conduct family support functions such as Farm Family Gatherings.

## Limitations

The study does have limitations, both to do with the method and also the conclusions that can be drawn from the findings. First the data were generated from self reports rather than records of actual communications and so may contain the biases associated with such reports, such as recall and social desirability. We sought to minimise recall accuracy and hence bias by asking about communications in general rather than over specific timeframes.

We do not know whether the network lists that we created were accurate or whether some relevant agencies may have been left off the lists. Such omission were unlikely, however, as the lists were created from independent input from three separate key informants per location. In addition, the nature of the data collection by face to face interview did generate considerable anecdotal data during which any key unlisted agencies would most likely have been named. Some non listed agencies were mentioned but only by a few respondents and these were not considered to be all that relevant as "network players". Also the data on links were not complete, as 22% of agencies overall did not participate in the survey and so did not provide their own information about their links. While this would have some effect on the results, the impact of this effect is difficult to ascertain. This problem was overcome to some extent as we used InDegree data and so we do have information about the links to all the 111 agencies across the four shires, but these come from the responses of the 87 participating agencies rather than from the 111.

The reasons for the different probability levels for giving and receiving information and the slightly higher mean effectiveness scores for receiving information over giving information are not clear. These may simply be an artefact related to the study power or because these were different questions on the survey with different psychometric properties. A response to "giving information" might focus the respondent more on their own effectiveness capabilities "to give", whereas response to "receiving information" might focus the respondent more on the effectiveness capability of the other party to "give to them". Another explanation may relate to expectations, where giving information to another agency may hold an expectation that the other agency will use that information effectively, while receiving information may hold that same expectation, but only about one's self. Detailed analysis of the nuances of cross sector agency communication was not within the scope of this study, but clearly improvements in communication between services sectors would benefit from such an understanding.

## Conclusion

Mental health program developers can use network information to see who are the most and least linked agencies and to establish a baseline on the rated frequency and effectiveness of these links. Highly linked agencies are "powerful" in network terms and can be used as the service point to add resources or support so that farmers are linked via these highly networked agencies to the professional health agents. In addition, these highly linked agencies can be influential in promoting change, such as to make service network improvements.

Networks that service the mental health needs of farming families should include agencies in close human service contact with them. This requires work to ensure that local service links are operational and effective and network descriptions provide a baseline for this work. The next phase of our work in this area involves the use of community mental health development officers to promote this network development, which includes the training of agricultural support workers in the early recognition of mental health distress in others and some basic first level responding skills.

## Competing interests

The authors declare that they have no competing interests.

## Authors' contributions

JF coordinated the network research component of the study, conducted the analysis and wrote the drafts of this paper. BK coordinated the overall study and contributed to drafts of this paper. SL and GP collected the network data and contributed to drafts of this paper. LF helped to design the study and contributed to drafts of this paper. All authors read and approved the final manuscript.

## Pre-publication history

The pre-publication history for this paper can be accessed here:



## Supplementary Material

Additional file 1 **Appendix**. Building Mental Health Awareness and Service Networks in Rural Australian Communities – A Service Delivery Evaluation. Click here for file
