# Supplementary material for: Service network analysis for agricultural mental health
Source: BMC Health Serv Res. 2009 May 29;9:87. doi: 10.1186/1472-6963-9-87 (PMC2700096; doi:10.1186/1472-6963-9-87)
Supplement: Additional file 1 — Appendix. Building Mental Health Awareness and Service Networks in Rural Australian Communities – A Service Delivery Evaluation. [file 1472-6963-9-87-S1.pdf]

Appendix 1. Survey instrument

Building Mental Health Awareness and Service Networks in Rural Australian Communities – A Service Delivery Evaluation

Service Network Analysis Survey

Interview #:____Name:_________________________________ Role:_______________________
Organisation:____Location/s: ________________
Phone:_____________________ Mobile: _______________________ Fax:__________________
Email: ____
Date:_____________________Code ________________

Listed on the scoring sheet are workers/ agencies involved in some way in the provision of services to farmers and farming families in the [INSERT LOCATION] area. We would like to know whether you are involved with these workers/ agencies in order to assist farmers or farm families experiencing mental, emotional or stress related problems.

A. INFORMATION EXCHANGE

We define information exchange as the process by which workers/ agencies communicate with each other about service provision for a client or a group of clients, in this case being farmers and farming families. Communication methods may include:

- Direct communication with the worker/ agency in question. Contact may be face-to-face, over the phone, by mail or by any electronic means.
- Through the joint attendance at meetings or formal/ informal gatherings where service and/or clients needs may be discussed with this worker/ agency.

A1.1 Do you ever give information to other workers/ agencies that is relevant to the provision of help when farmers or farming families are experiencing mental, emotional or stress related problems?

Yes ☐ No or don't know ☐ (if no or don't know go to A2.1)

A1.2 Which workers/ agencies on the list do you give this information to?

For each of the workers or agencies that you have named

A1.3 In general, how often do you give this type of information <u>to</u> this worker/ agency (*choose the most frequent*)?

weekly	5
monthly	4
3 monthly	3
half yearly	2
less often than half yearly	1

The next question asks about the effectiveness of this communication. By effectiveness, we mean the extent to which the intent of communication is achieved, which involves the capabilities of both the sender & receiver

A 1.4. In general, how would you rate the effectiveness of the communication of this information <u>to</u> this worker/ agency?

very good	5
good	4
adequate	3
poor	2
very poor	1

A2.1 Do you ever receive information from other workers/ agencies that is relevant to the provision of help when farmers or farming families are experiencing mental, emotional or stress related problems?

Yes ☐ No or don't know ☐ (if no or don't know go to B)

A2.2 Which workers/ agencies on the list do you receive this information from?

For each of the workers or agencies that you have named

A2.3 In general, how often do you receive this type of information from this worker/ agency (*choose the most frequent*)?

weekly	5
monthly	4
3 monthly	3
half yearly	2
less often than half yearly	1

A2.4 In general, how would you rate the effectiveness of the communication of this information from this worker/ agency?

very good	5
good	4
adequate	3
poor	2
very poor `	1

B. RECOMMEND

By recommend we mean making a suggestion through to actively and/or formally making a referral between a client and another worker/ agency regardless of whether the client acts on the recommendation. The process of recommendation by one agency to another may include the following methods:

- The formal process of first-hand referral by letter or telephone ie. via the use of official forms or documentation.
- A less formal recommendation through a spoken or written suggestion.

If a list of helping agencies is given to a client then we do not consider that to be a recommendation unless this includes a recommendation about seeing a particular worker or agency.

B1 Do you ever recommend that a farmer or farming family see other workers/ agencies for help when they are experiencing mental, emotional or stress related problems?

Yes ☐ No ☐ (if no go to C)

B2 Which workers/ agencies on the list do you make recommendations to?

For each of the workers or agencies that you have named

B3. In general, how often would you make a recommendation to a farmer or farming family about seeing this worker/ agency (*choose the most frequent*)?

weekly	5
monthly	4
3 monthly	3
half yearly	2
less often than half yearly	1

C. WORKING TOGETHER IN OTHER WAYS

Working together in other ways refers to the organisation of resources (staff, funding, equipment etc) and the development of strategies to benefit farmers and farming families who are experiencing mental, emotional or stress related problems, such as organising Farm Family Gatherings or lobbying to increase support services in the area.

C1 Do you ever work together in other ways with other workers/ agencies to benefit farmers or farming families who are experiencing mental, emotional or stress related problems?

Yes ☐ No or don't know ☐ (if no or don't know go to D)

C2. Which workers/ agencies on the list do you work together with in other ways?

For each of the workers or agencies that you have named

C3. In general, how often do you and this worker/ agency work together in other ways (*choose the most frequent*)?

weekly	5
monthly	4
3 monthly	3
half yearly	2
less often than half yearly	1

C4. In general, how would you rate the effectiveness of how you and this worker/ agency work together in other ways?

very good	5
good	4
adequate	3
poor	2
very poor	1

D. NETWORK

D1. Do you meet regularly as a group or network with other agencies in the (NAME) area?

Yes ☐ No ☐ (if no go to E)

D2. How often do you meet as a group or network?

weekly	5
monthly	4
3 monthly	3
half yearly	2
less often than half yearly	1

D3. In general, how would you rate the effectiveness of the group or network?

very good	5
good	4
adequate	3
poor	2
very poor	1

E. NEED

E1. In recent times what percentage of the farmers or farming family members that you saw do you consider had mental, emotional or stress related problems that would benefit from some sort of help?
